# Optimization of the Extraction Process to Obtain a Colorant Ingredient from Leaves of *Ocimum basilicum* var. *purpurascens*

**DOI:** 10.3390/molecules24040686

**Published:** 2019-02-14

**Authors:** Filipa Fernandes, Eliana Pereira, Miguel A. Prieto, Ricardo C. Calhelha, Ana Ćirić, Marina Soković, Jesus Simal-Gandara, Lillian Barros, Isabel C. F. R. Ferreira

**Affiliations:** 1 Centro de Investigação de Montanha (CIMO), Instituto Politécnico de Bragança, Campus de Santa Apolónia, 5300-253 Bragança, Portugal; filipafernandes1994@outlook.com (F.F.); eliana@ipb.pt (E.P.); mprieto@ipb.pt (M.A.P.); calhelha@ipb.pt (R.C.C.); 2 Nutrition and Bromatology Group, Department of Analytical and Food Chemistry, Faculty of Food Science and Technology, University of Vigo—Ourense Campus, E-32004 Ourense, Spain; jsimal@uvigo.es; 3 University of Belgrade, Department of Plant Physiology, Institute for Biological Research “Siniša Stanković”, Bulevar Despota Stefana 142, 11000 Belgrade, Serbia; rancic@ibiss.bg.ac.rs (A.Ć.); mris@ibiss.bg.ac.rs (M.S.)

**Keywords:** natural colorants, anthocyanins, *Ocimum basilicum* var. *purpurascens* leaves, red rubin basil, Heat-Assisted Extraction, extraction optimization

## Abstract

Heat-Assisted Extraction (HAE) was used for the optimized production of an extract rich in anthocyanin compounds from *Ocimum basilicum* var. *purpurascens* leaves. The optimization was performed using the response surface methodology employing a central composite experimental design with five-levels for each of the assessed variables. The independent variables studied were the extraction time (*t*, 20–120 min), temperature (*T*, 25–85 °C), and solvent (*S*, 0–100% of ethanol, *v/v*). Anthocyanin compounds were analysed by HPLC-DAD-ESI/MS and the extraction yields were used as response variables. Theoretical models were developed for the obtained experimental data, then the models were validated by a selected number of statistical tests, and finally, those models were used in the prediction and optimization steps. The optimal HAE conditions for the extraction of anthocyanin compounds were: *t* = 65.37 ± 3.62 min, *T* = 85.00 ± 1.17 °C and *S* = 62.50 ± 4.24%, and originated 114.74 ± 0.58 TA mg/g of extract. This study highlighted the red rubin basil leaves as a promising natural matrix to extract pigmented compounds, using green solvents and reduced extraction times. The extract rich in anthocyanins also showed antimicrobial and anti-proliferative properties against four human tumor cell lines, without any toxicity on a primary porcine liver cell line.

## 1. Introduction

Consumers’ interest in food quality has been increasing, selecting foods with health benefits. Colour is the main organoleptic attribute in the selection and acceptance of foods [1,2]. Some vegetable matrices are composed by natural pigments, attracting much attention from the scientific community and leading to studies to characterize these compounds and explore their subsequent application, not only in the food industry as natural colorants, but also in the pharmaceutical sector, as antioxidants [3,4,5].

Anthocyanins are natural pigment studied worldwide; however, when these compounds are incorporated in food products, there are several intrinsic and extrinsic factors that affect and influence their stability [6]. The pH is an important parameter, because it is crucial in the determination of the anthocyanin colour, which shows a significant pigmentation variability. In aqueous medium, they are red at pH = 1–3, colourless at pH = 4–5, purple at pH = 6–7, blue at pH = 7–8, and yellow at pH = 8–9 [3,7]. Other variables to be taken into account in anthocyanins’ stability are the handling and storage temperature, the chemical composition of target products (presence of enzymes, proteins, metal ions and even other flavonoids), and exposure to light and oxygen [3].

The use of anthocyanins as food colorants is approved in several countries [8] and according to the Regulation (EU) nr 1129/2011 of the Commission of 11 November 2011, their application is authorised in numerous food products and processes, such as cured cheeses and cheese products of red marbled paste, vegetables in vinegar, oil or brine (except olives), jams, jellies and marmalades, fruit-flavored breakfast cereals, fish pastes and crustaceans, pre-cooked crustaceans, and smoked fish, among other products. The acceptable daily intake (ADI) is not regulated, which means that sufficient quantity can be added to food products to achieve the desired coloration effect [9].

Anthocyanins can be found in numerous natural sources, especially in fruits, cereals, leaves, flowers, and roots, such as in the leaves of *Ocimum basilicum* var. *purpurascens* (red rubin basil) [10]. Red rubin basil belongs to the *Lamiaceae* family, being a variety of *Ocimum basilicum*, and is used not only as an ornamental plant, but also in traditional medicine [10,11,12]. 

In order to apply sustainable extraction methodologies at an industrial level, mathematical studies are performed to maximize the extraction of compounds from natural matrices [13,14]. The patterns of the response variables of the extraction method, such as processing temperature, time and solvent [13,14] can be evaluated using the response surface methodology (RSM). This technique allows to save time, reagents and reduce the operational costs, meanwhile increases the efficiency of the optimization process. Aiming to promote the applicability of natural pigments present in the *Ocimum basilicum* var. *pupurascens* leaves at an industrial level, this work optimized the HAE extraction of anthocyanin compounds, particularly cyanidin and pelargonidin derivatives using Response Surface Methodology (RSM).

## 2. Results

### 2.1. Response Criteria for the RSM Analysis

**Figure 1:**
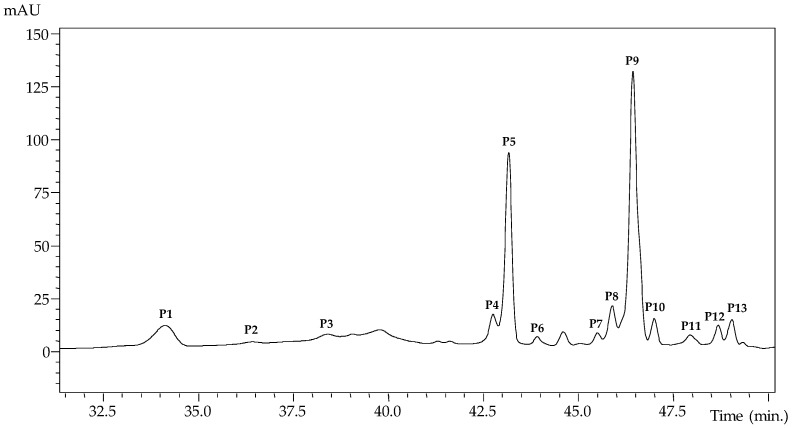
HPLC profile of anthocyanin molecules found in red rubin basil leaves extract obtained in the data set number 18 (as described in Table 2).

The HPLC anthocyanin profile of the red rubin basil leaves extract from experimental run number 18 is shown in Figure 1. Up to 13 anthocyanin compounds were identified (Table 1) based their chromatographic characteristics (UV-Vis, mass spectral fragmentation patterns) and literature information [15,16]. 

**Figure 2:**
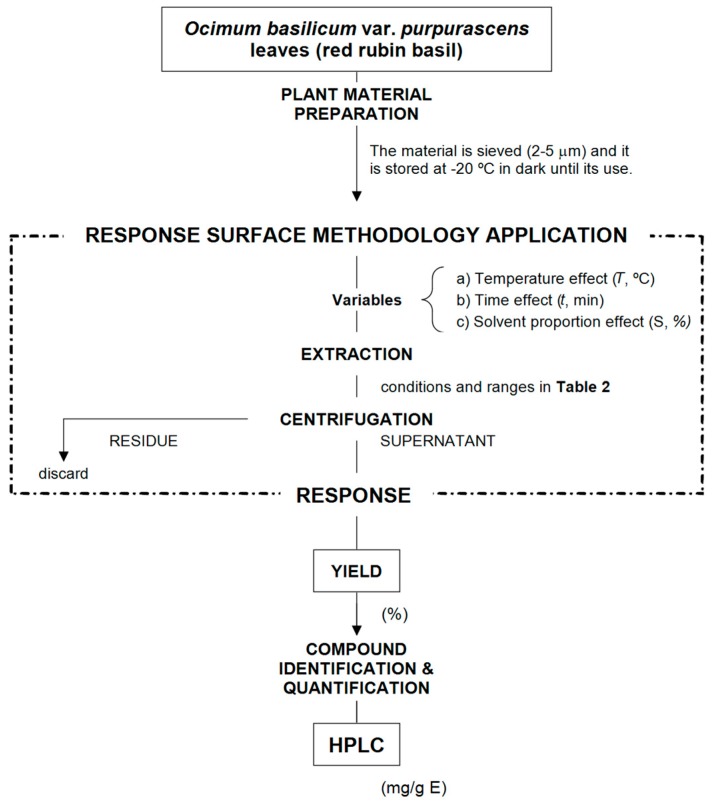
Diagram of the different steps carried out for optimizing the conditions that maximize the extraction responses of the anthocyanin compounds and the total extracted residue (Yield, %).

Figure 2 shows a summary of the diverse stages used for optimization procedure, in order to recover the anthocyanin compounds from the red rubin basil leaves. The experimental values of the 28 experimental runs of the circumscribed central composite design (*CCCD*) design are presented in Table 2.

The content in individual (**P1** to **P13**) and grouped (TAC – total anthocyanin compounds) anthocyanin compounds were used as criteria to maximize their content and to optimize the extraction conditions of HAE from red rubin basil leaves under RSM assessment. The values of the extraction yield were also considered, and ranged from 13.22 to 41.00%, with the experimental runs no. 16 and 21, respectively (Table 2). In total, 15 response variables are taking into account for the optimization processes.

### 2.2. Theoretical Response Surface Models

Evaluating the precision of theoretical models to predict and comprehended the effects of independent variables in some response variable is necessary. This, as in many research fields, is achieved by fitting these models to the experimental values. In this study, a non-linear algorithm (least-squares estimates) has been used to adjust the response values (Table 2) to a second order polynomial model. The estimated coefficient values obtained from the polynomial model of Equation (1) and the coefficient of correlation (R^2^) for each parametric response of the extraction process are shown in Table 3.

(1) $$y=b_{0}+\;\overset{n}{\underset{i=1}{\sum }}\;b_{i}X_{i}+\;\overset{n-1}{\underset{\begin{array}{c} i=1\\ j>i \end{array}}{\sum }}\;\overset{n}{\underset{j=2}{\sum }}\;b_{ij}X_{i}X_{J}+\;\overset{n}{\underset{i=1}{\sum }}\;b_{ii}X_{i}^{2}$$

The parametric values obtained, not only it allows to translate response patterns, it also helps to undestand the complexity of the possible interactions between variables. However, some of the parameters of Equation (1) whose coefficients were non-significant (*ns*) at a 95% confidence level (α = 0.05) were not used for building the model. By means of the statistic lack of fit it is possible to prove the adequacy of the obtained models and in this way it was demonstrated that a considerable improvement was not achieved by means of the inclusion of the statistically *ns* parametric values. Each of the 15 assessed responses can be seen in models in Table 4 getting in all cases *R*^2^ coefficients higher than 0.92 (Table 3). According to this value, it can be said that the percentage of variability of each response can be explained by the model.These workable models were applied in the subsequent prediction and optimization steps, with a good agreement between the experimental and predicted values, which indicates that the variation is explained by the independent variables.

Although the obtained model coefficients (Table 3) cannot be associated with physical or chemical significance and are empirical, they can however be used to predict the results of untested extraction conditions [17]. As the effect sign marks the performance of the response, if a factor has a positive effect, the response is higher at the high level. On the other hand, the response is lower at the high level when a factor has a negative effect. Therefore, the weight of the corresponding variable will be more important the higher the absolute value of a coefficient. Certain characteristics relating to the general effects of the variables based on mathematical expressions can be observed in Table 4. The relevance of the significant parametric values can be order as a function of the variables involved in a decreasing form as *S* > *t* >> *T*. Previous authors that work with similar matrices [14], have concluded that the most relevant variable on the HAE extraction of bioactive compounds is *S*. As for the study of the linear, quadratic, and interactive parametric effects of the developed equations, it allowed to conclude that all these parameters play an important and significant role in all evaluated responses. For the linear effect, the variables *S* and *t* had strong values, while the effect of *T* was less important in almost all cases. All independent variables had moderate quadratic or nonlinear effects. As for the interactions of the variable (*tT*, *TS* and *tS*), these were of minor importance. The results obtained were represented in the response surface plots that can be seen below so that in this way one can see in a more obvious way the combined effects as well as to be able to visually describe the tendencies of extraction. The optimal HAE conditions, that maximize their retrieval from red rubin basil leaves, are presented in Table 3.

### 2.3. Final Effects of the Studied Conditions of HAE on the Target Responses and Optimal Values that Maximize the Responses

**Figure 3:**
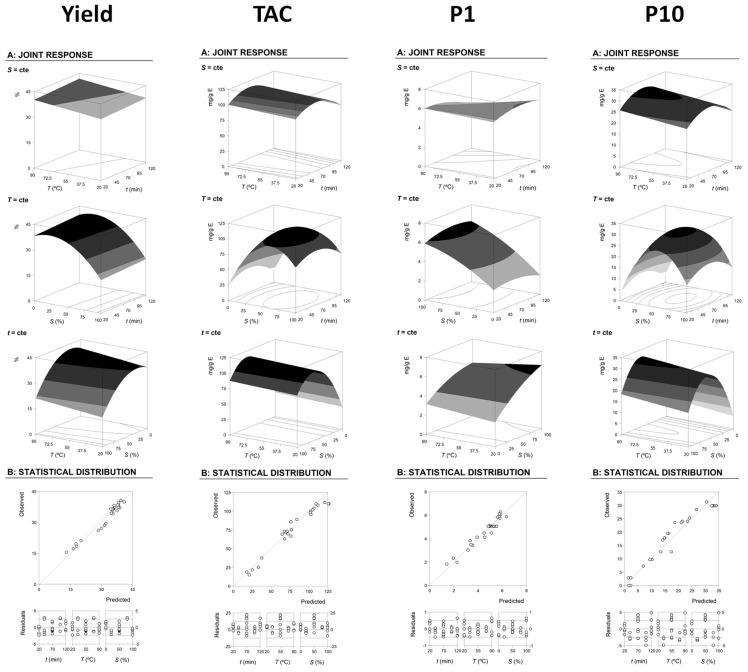
Illustrative representation of the extraction yield and grouped anthocyanin compounds (total anthocyanin acids, total flavonoids and total anthocyanin compounds) responses. The part A shows the 3D description as a function of each independent variable. The surfaces were constructed using the values presented in Table 3 and described by Equation (1). In each graph, the excluded variable was positioned at the optimum of their experimental domain (Table 3). Part B shows a summary of the goodness of fit using the observed/predicted and the residual distribution plots as a function of each variable.

Figure 3 shows the response surface plots of extraction yield, *TAC* and two other representative anthocyanins extracted (**P1** and **P10**), as well as their statistical analysis. Inspecting the given surface plots of the extraction yield (Figure 3), it is conceivable to confirm that the measure of removed material increments to an ideal point and afterward, by and large, it diminishes as a component of the included variables. Subsequently, the ideal values can be found similar to a solitary point, which permits figuring the extraction conditions that lead to the most extreme flat out. This behaviour is common to almost all responses, allowing us to determine the conditions that maximize the responses. In consequence, the ideal extraction values for the reactions shown in Figure 3 were determined for the HAE conditions (Table 3), as summarized below:

For yield, the optimal HAE conditions were: *t* = 120.00 ± 2.62 min, *T* = 85.00 ± 7.72 °C and 23.23 ± 0.91% of ethanol (*v*/*v*), and produced 41.77 ± 1.59%. 

For TAC, the optimal HAE conditions were: *t* = 65.37 ± 3.62 min, *T* = 85.00 ± 1.17 °C and 62.50 ± 4.24% of ethanol (*v*/*v*), and produced 114.74 ± 0.58 mg/g of E. 

For **P1**, the optimal HAE conditions were: *t* = 81.06 ± 2.08 min, *T* = 25.00 ± 1.73 °C and 100.00 ± 1.58% of ethanol (*v*/*v*), and produced 6.56 ± 0.31 mg/g of E. 

For **P10**, the optimal HAE conditions were: *t* = 70.00 ± 1.42 min, *T* = 85.00 ± 2.46 °C and 56.85 ± 0.94% of ethanol (*v*/*v*), and produced 31.63 ± 2.42 mg/g of E. 

**Figure 4:**
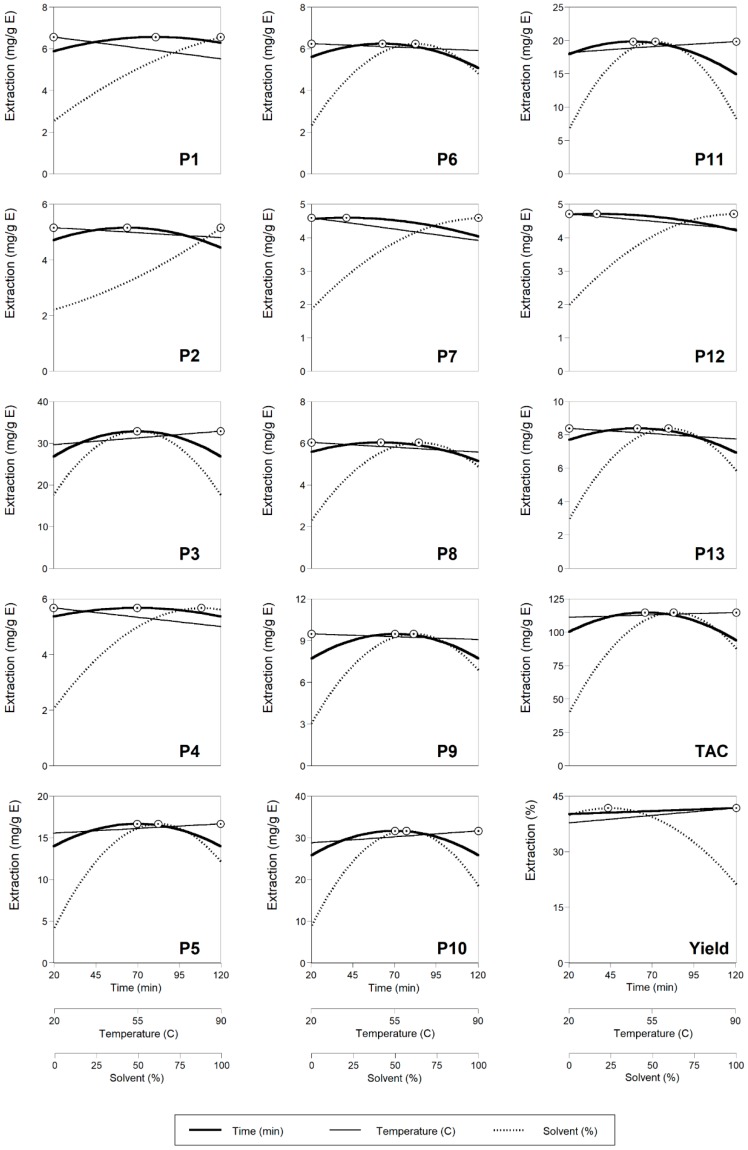
2D graphical response of the effects of the independent variables on the extraction of anthocyanin compounds from red rubin basil leaves (see Figure 1 for peak identification). Dots (ʘ) represent the optimal values. In each plot, each independent variable was positioned at the optimal value of the other two variables (Table 3).

It is well-known that the utilization of high values of ethanol in the solvent, increases the extraction of bioactive compounds from plant materials [13]. The effects of the independent variables on the extraction of individual anthocyanin compounds from red rubin basil leaves are represented in 2D in Figure 4. The processing conditions that generated optimal response values (ʘ) are numerically described in Table 3. The identified anthocyanin compounds were organized as a function of the maximum amount achieved (mg/g of extract) in a decreasing order as follows: **P3** (32.85) > **P10** (31.63) >> **P5** (16.66) >> **P9** (9.47) > **P11** (9.28) > **P13** (8.38) > **P1** (6.56) > **P6** (6.24) > **P8** (6.03) > **P4** (5.67) > **P2** (5.15) > **P7** (4.59) > **P2** (4.71).

The greater extraction values achieved under these optimized conditions highlight the suitability of HAE with RSM as an innovative process to recover a greater amount of anthocyanin compounds from red rubin basil leaves using shorter processing times and greener solvents.

### 2.4. Clustering of Anthocyanin Compounds According to the HAE Conditions that Maximize their Extraction

**Figure 5:**
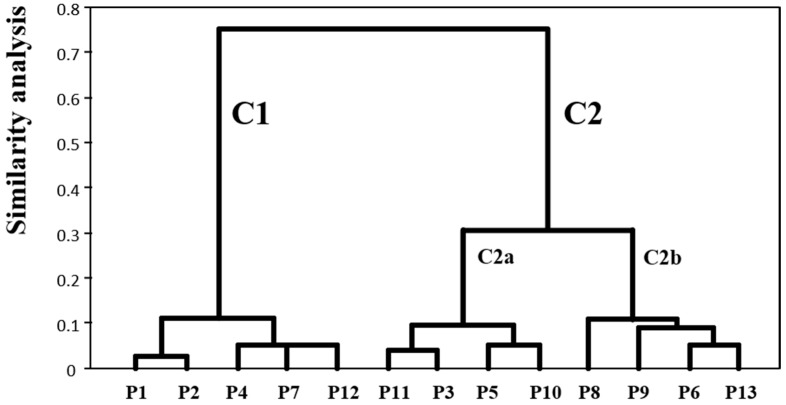
HCA dendrogram of anthocyanin compounds according to the HAE conditions that maximize their extraction from red rubin basil leaves.

The maximum values for the response values of the different anthocyanin compounds and their concentrations if extracted under the optimal HAE conditions of the other compounds (Table 3) are presented in Table 5. The values of subparagraph (B) is the ratio of the optimum value of each compound between the maximum of the other compounds. When two compounds show values of 100%, i.e., the coefficient is 1, under the same conditions of HAE means that the optimal response value for both is in the same conditions. As example, the compounds **P1**, **P2**, **P4**, **P7** and **P12** were clustered in C1 under the same HAE conditions (Figure 5). By cons, if the coefficient is different from 1, it means that the conditions that are optimal for the extraction of a compound are not for the other (compounds **1** and **13**).

In Table 5 it can be observed the formation of different groups of compounds of anthocyanin with maximum response values in conditions of HAE extraction similar. The division in these groups was made possible by the complete data set of Table 5 and by performing a multi objective optimization problem using an appropriate clustering algorithm. The results of Hierarchical Cluster Analysis (HCA) are presented in Figure 5. In the HCA dendrogram, the shorter distance between compounds, the higher similarity in terms of conditions that favour their extraction. Moreover, compounds belonging to the same group are better extracted under similar HAE conditions. Two significant clusters (C1 and C2), being the C2 divided in turn into 2 subgroups (a and b). Other less important subgroups were created, but they can be considered as a residual noise produced by the algorithm.

Cluster C1 included the compounds **P1**, **P2**, **P4**, **P7** and **P12**. The extraction of these compounds for maximize by medium *t*, high *S* and low/high *T* (Table 3 and Figure 3). The subgroups were mainly differentiated by the *T* values.

Cluster C2 included all other compounds **P11**, **P3**, **P5**, **P10**, **P8**, **P9**, **P6** and **P13**, which were subdivided in C2a and C2b. For maximizing the extraction of the compounds in C2a low *T* and medium *S* was used. On the other hand, the compounds in C2b was maximized when using high *T* and medium *S*.

Although it was expected that if the compounds have similar chemical characteristics also would have similar HAE conditions, the HCA analysis was an interesting and innovative approach in the field of extraction of high added-value compound from natural sources since this analysis highlighted suitable HAE conditions for maximize the simultaneous recovery of specific groups of compounds from red rubin basil leaves.

### 2.5. Dose-Response Analysis of the Solid-to-Liquid Effect at the Optimum Conditions 

Thanks to the precise results obtained by HPLC, the *S/L* effect was tested under the optimal conditions provided for each extractive technique by the polynomial models, using the amount of anthocyanin as response. As confirmed by the preliminary results (data not shown), the maximum experimental value is close to 30 g/L, since at higher values of *S/L* it is observed experimental stirring, so an experiment was designed for each extractive process in which to check the *S/L* behaviour at values between 1 and 30 g/L. The obtained results are consistent with previous responses. It was observed that the effect caused by the *S/L* ratio follows a simple linear model with an intercept, and that this model follows a slightly decreasing pattern proportional to the increase of *S/L* in all the assays. However, that pattern, explained by the parametric coefficient of the slope, was non-significant with a confidence interval level of 95 % (α = 0.05) and the decreasing effect was not taken into account for further analysis. In conclusion, it can be affirmed that the increase in the *S/L* ratio has very little effect on the *TAC* extraction, besides that saturation effects were not observed at any value below 30 g/L.

### 2.6. Evaluation of the Colorant Potential of the Extract Rich in Anthocyanin Compounds Obtained under Optimum Conditions from Leaves of O. basilicum var. purpurascens 

The results of the chromatic analysis in the CIE L*a*b* colour space of the extract rich in anthocyanins present in the leaves of *O. basilicum* var. *purpurascens* are shown in Table 6. The colour of the pigmented extract showed an L* value, lightness (0 to 100), of 20.5 ± 0.5; and in parameters a* (colour intensity from green to red (−120 to 120)) and b* (colour is evaluated at the intensity level from blue to yellow (−120 to 120)), the values were 33.0 ± 0.1 and 8.2 ± 0.4, respectively. 

For a better understanding of the colour values, these were converted to RGB values and the colour obtained from the extract, red-berry, can be visualized. These results can be justified by the presence of anthocyanin compounds in the extract, which, in addition to having darker shades, are also characterized by blue, red and purple tones. The concentration of total anthocyanin compounds, obtained in the optimized extract, was similar to that predicted by the model.

### 2.7. Evaluation of the Bioactive Properties of the Extract Rich in Anthocyanin Compounds Obtained under Optimal Conditions from Leaves of O. basilicum var. purpurascens

#### 2.7.1. Antimicrobial Activity

Table 7 shows the results of the antimicrobial activity obtained from the extract rich in anthocyanins present in the leaves of *O. basilicum* var. *purpurascens*. The results demonstrate antibacterial activity of the pigmented extract for all microorganisms’ strains. In this way, the best results are obtained against *Bacillus cereus* (*B.c*.) (MIC = 0.037 mg/mL; MBC = 0.075 mg/mL) and *Escherichia coli* (*E.c.*) (MIC = 0.037 mg/mL; MBC = 0.075 mg/mL) strains. However, the pigmented extract also showed a high activity against *Listeria monocytogenes* (*L.m.*) (MIC = 0.05 mg/mL; MBC = 0.075 mg/mL), *Staphylococcus aureus* (*S.a.*), *Enterobacter cloacae* (*En.cl.*) (MIC = 0.075 mg/mL, MBC = 0.15 mg/mL), and *Salmonella typhimirium* (*S.t.*) (MIC = 0.15 mg/mL; MBC= 0.30 mg/mL).

Regarding antifungal activity, the extract showed a high potential against most of the tested fungi. *Aspergillus ochraceus* (*A.o.*) was the most susceptible species to the extract (MIC = 0.002 mg/mL; MFC = 0.075 mg/mL); however, no antifungal activity was observed against *Penicillium verrucosum* var. *cyclopium* (*P.v.c.*) (MIC = 0.30 mg/mL; MFC = 0.45 mg/mL). These results indicated a promising antimicrobial activity, and this can be explained due to the high concentration of anthocyanin compounds that have a high antimicrobial potential [18].

#### 2.7.2. Cytotoxic Activity

Table 8 shows the results obtained in the cytotoxicity evaluation assays in extracts rich in anthocyanin compounds, obtained through optimal extraction conditions. The extract exhibited anti-proliferative capacity in HeLa (GI_50_ = 213 ± 9 μg/mL) and HepG2 (GI_50_ = 198 ± 9 μg/mL) tumour cell lines. 

These results may also be explained by the high levels of anthocyanin compounds present in the extract, since these molecules have been described, by several authors, as a potential anti-proliferative agent in tumor cell lines [19]. Regarding the assay performed on primary non-tumor cell culture (PLP2), the extract evidenced the absence of toxicity up to the maximal tested concentration (GI_50_ > 400 μg/mL).

## 3. Materials and Methods 

### 3.1. Samples

*Ocimum basilicum* var. *purpurascens* (Lamiaceae) variety was obtained in Cantinho das Aromáticas, Vila Nova de Gaia, Portugal. The samples acquired were planted to grow in greenhouse at the Polytechnic Institute of Bragança and then collected (September 2017). The fresh leaves were separated through a mechanical procedure, posteriorly lyophilized (FreeZone 4.5, Labconco, Kansas City, MO, USA), reduced to a fine and homogeneous dried powder (~20 mesh) and stored protected from light and heat.

### 3.2. Heat-Assisted Extraction

Heat-Assisted Extraction (HAE) was performed in a water reactor agitated internally with a Cimarec^TM^ Magnetic Stirrer at a constant speed (~500 *rpm*, Thermo Scientific, San Jose, CA, USA), following a procedure previously performed by Roriz et al. [20]. The powdered samples (300 mg) were extracted with solvent (20 mL of ethanol/water) under diverse conditions, as previously defined by the established RSM plan (Table 2). The ranges of the experimental design were: time (*t* or *X*_1_, 20 to 120 min), temperature (*T* or *X*_2_, 25 to 85 °C) and ethanol content (*S* or *X*_3_, 0 to 100%). The solid-to-liquid ratio (*S/L*) was kept at 15 g/L for all conditions.

When all the individual extraction conditions were carried out, the samples were immediately centrifuged (4750× *g* during 20 min at 10 °C) and filtered (paper filter Whatman n° 4) to eliminate the non-dissolved material. The supernatant was collected and divided in two portions for HPLC and extraction yield analysis. The portion separated for HPLC analysis (2 mL) was filtered through a LC filter disk (0.22 µm), whereas the portion for the extraction yield determination (5 mL) was dried at 105 °C during 48 h and thereafter weighted.

### 3.3. Calculation of the Extraction Yield

The extraction yields (%) were calculated based on the dry weight (crude extract) obtained after evaporation of the solvent. In all cases, the filtrates were concentrated at 35 °C in a rotary evaporator (Büchi R-210, Flawil, Switzerland) under reduced pressure and the aqueous phase was then lyophilised to obtain a dried extract. 

### 3.4. Chromatographic Analysis of Anthocyanin Compounds

The samples were analysed using Dionex Ultimate 3000 UPLC (Thermo Scientific, San Jose, CA, USA) coupled to a diode de array detector (chromatograms recorded at 520 nm) and to a Linear Ion Trap LTQ XL mass spectrometer (Thermo Finnigan, San Jose, CA, USA) equipped with an ESI source working in positive mode, following a procedure previously reported [21]. Quantitative analysis was performed using a calibration curve obtained using cyanidin-3-glucoside (y = 97,787x − 743,469; *R*^2^ = 0.9993) and pelargonidin-3-glucoside (y = 43,781x − 275,315; *R*^2^ = 0.9989) and results were expressed in mg per g of extract (mg/g E).

### 3.5. Experimental Design, Modelling and Optimization

#### 3.5.1. Experimental Design

A RSM of five-level *CCCD* of 28 runs with 6 replicated values at centre points was applied to optimize the HAE conditions for the extraction of anthocyanin compounds. Coded and natural values of the independent variables *X*_1_ (processing time (*t*), min), *X*_2_ (temperature (*T*), °C) and *X*_3_ (solvent (*S*), % of ethanol, *v/v*) are presented in Table 1.

#### 3.5.2. Mathematical Modelling

The response surface models were fitted by means of least-squares calculation using the following second-order polynomial equation with interactive terms (Equation (1)). In this equation, *Y* represents the dependent variable (response variable) to be modelled, *X_i_* and *X_j_* are the independent variables, *b*_0_ is the constant coefficient, bi is the coefficient of linear effect, *b_ij_* is the coefficient of interaction effect, *b_ii_* is the coefficient of quadratic effect, and n is the number of variables. The extraction yield and the individual and grouped anthocyanin compounds, 13 individual compounds plus the total anthocyanin content (*TAC*), were used as dependent variables.

#### 3.5.3. Maximization of the Responses

For the extraction yield and the recovery of phenolic compounds responses, a *simplex* method was used for maximize the models developed of Equation (1) [22]. In all cases, restrictions were added to limit the values of the conditions assessed.

### 3.6. Gropping the Responses by Cluster Analyses

A cluster analysis was performed to group the anthocyanin compounds according to the extraction conditions that maximize their response values using the Excel add-in “XLSTAT 2016” (Addinsoft, Barcelana, Spain). A comparative agglomerative hierarchical clustering analysis (HCA) with automatic truncation based on entropy and Pearson correlation coefficient were used for clustering (similarity analysis).

### 3.7. Fitting Procedures and Statistical Analysis

Fitting procedures, coefficient estimates and statistical calculations were performed as previously described by Prieto and Vázquez [23]. In brief: (a) fitting procedure by nonlinear least-square (quasi-Newton) as provided by the Excel add-in “Solver”; (b) coefficient intervals determination by the Excel add-in “SolverAid“; and (c) the model consistency by common statistical tests for each model developed: (i) the Fisher F-test (*α* = 0.05); (ii) parametric assessment by the Excel add-in “;SolverStat“; (iii) the determination of *R*^2^.

### 3.8. Preparation of the Extract Rich in Anthocyanin Compounds Obtained under Optimum Conditions from the Leaves of O. basilicum var. purpurascens

For the preparation of an extract rich in anthocyanin compounds, extraction from the leaves of *O. basilicum* var. *purpurascens* was performed, following the previously optimized procedure (Table 1). The samples (300 mg) were placed together ethanol/water (20 mL, 55:45, *v/v*) acidified with 0.25% citric acid (pH = 3) in a glass vial with a stopper. The extraction followed established conditions of temperature (*T* = 72 °C) and time (60 min). After the procedure described, the sample was centrifuged (Centurion K24OR, West Sussex, UK) at 5000 rpm for 5 min at 10 °C. They were then filtered through filter paper (Whatman n° 4) to remove suspended solids. The ethanol fraction was removed at a temperature of 35 °C and the aqueous fraction obtained was frozen and lyophilized (FreeZone 4.5), affording an extract rich in anthocyanin compounds. The lyophilized extract was stored away from the light for further analysis.

### 3.9. Evaluation of the Colorant Potential of the Extract Rich in Anthocyanin Compounds Obtained under Optimum Conditions from the Leaves of O. basilicum var. purpurascens

The evaluation of the colorant potential of the extract was carried out by measuring the colour and the measurement of the colouring compounds by chromatography, in order to corroborate the data provided by the MRS. The colour was measured using a colorimeter (model CR-400, Konica Minolta Sensing, Inc., Osaka, Japan) with an adapter for granular materials (model CR-A50), according to a procedure described by Pereira et al. [24]. The measurements were made in the CIE L*a*b* colour space, using the illuminant C and a diaphragm aperture of 8 mm. Data were processed with the “Spectra Magic Nx” (version CM-S100W 2.03.0006 software, Konica Minolta). Quantitation of anthocyanin compounds was accomplished by chromatography using an HPLC-DAD-ESI/MS system as described in Section 3.4.

### 3.10. Evaluation of the Bioactive Properties of the Extract Rich in Anthocyanin Compounds Obtained under Optimal Conditions from the Leaves of O. basilicum var. purpurascens.

#### 3.10.1. Antimicrobial Activity

The antimicrobial activity was evaluated using the methodology described by Carocho et al. [25]. Gram-negative (*Enterobacter cloacae* (American Type Culture Collection (ATCC) 35030), *Escherichia coli* (ATCC 35210) and *Salmonella typhimurium* (ATCC 13311)) and Gram-positive (*Bacillus cereus* (clinical isolate), *Listeria monocytogenes* (NCTC (National collection of type cultures) 7973) and *Staphylococcus aureus* (ATCC 6538)) bacteria strains were used. For the calculation of the minimum inhibitory (MIC) and minimum bactericidal (MBC) concentrations, the microdilution method was applied and the results were expressed in mg/mL.

For the antifungal activity, a procedure previously described by Carocho et al. [25] was followed. *Aspergillus fumigatus* (ATCC 1022), *Aspergillus niger* (ATCC 6275), *Aspergillus ochraceus* (ATCC 12066), *Penicillium funiculosum* (ATCC 36839), *Penicillium ochrochloron* (ATCC 9112) and *Penicillium verrucosum* var. *cyclopium* (food isolate) were used. Minimum inhibitory concentration (MIC) and minimum fungicidal concentration (MFC) were also determined by using the microdilution method and the results were also expressed in mg/mL. 

#### 3.10.2. Cytotoxic Activity

The evaluation of the cytotoxic potential of the extract rich in anthocyanin compounds was performed by the Sulfarodamine B (SRB) assay previously described by Barros et al. [26] MCF-7 (breast carcinoma), NCI-H460 (lung carcinoma), HeLa (cervical carcinoma) and HepG2 (hepatocellular carcinoma) were used as human tumor cell lines. For the hepatotoxicity assay, the extract rich in anthocyanin compounds was tested in a primary non-tumor cell culture obtained from porcine liver (PLP2).

Ellipticine (Sigma-Aldrich, St. Louis, MO, USA) was used as the positive control and the results were expressed as GI_50_ values (sample concentration that inhibits the growth of cells by 50%), and expressed in μg/mL.

## 4. Conclusions

Colorants are one of the most important additives in terms of marketing, because their presence in food products is considered the principal factor influencing customer choice. To the authors’ best knowledge, the potential industrial use of the anthocyanin compounds from red rubin basil leaves have not been explored previously. In such a context, the present work presents a new rapid method to extract anthocyanin compounds from red rubin basil leaves. RSM and other mathematical strategies were successfully employed to optimize extraction conditions that maximize the anthocyanin recovery to produce a rich extract with potential for industrial application as a natural colouring additive. 

The scientific literature shows clear evidence that extraction procedures of target compounds from plant-based products, must be assessed individually. Therefore, a nonstop effort needs to be performed, because agro-industrial and food sectors are looking for byproduct valorisation into added-value products. However, in order to take full advantage of the technological advances, the extraction conditions need to be optimized. Mathematical solutions, such as RSM tools, could increase the efficiency and profitability of the process and help to change conventional extraction approaches. 

In this study, the suitability of HAE for extracting anthocyanin compounds from red rubin basil leaves was demonstrated and the variables of *t*, *T* and *S* were combined in a five-level *CCCD* design coupled to RSM for optimization. According to the results, a good agreement between experimental and theoretical results was observed. In general, the recovery of anthocyanin compounds was maximized when high temperatures, high ethanol concentrations and medium extraction times were applied, validating this Heat-Assisted Extraction. 

The colour analysis in the pigmented extract revealed interesting values, showing dark tones, more directed to a red tonality. It was also evident the antimicrobial and anti-proliferative potential against several strains and tumour cell lines, respectively, without presenting toxicity for non-tumor cells.

These results should promote interest in conducting further studies on *O. basilicum* varieties, highlighting the potential of ruby red basil as a potential source of natural and bioactive ingredients with application in several industrial factors, namely in the food and pharmaceutical areas.

## Figures and Tables

**Table 1:** Retention time (Rt), wavelengths of maximum absorption in the UV-Vis region (λ_max_), and tentative identification of anthocyanin compounds in *O. basilicum* var. *purpurascens* (mean ± SD).

Peak	Rt (min)	λ_max_ (nm)	[M + H]^+^	Main Fragment ESI- MSn [Intensity (%)]	Tentative Identification
**P1**	34.1	520	919	757(49),449(6),287(13)	Cyanidin-3-(*p*-coumaroyl-6′-caffeoyl)sophoroside isomer 1 ^A^
**P2**	36.4	520	919	757(49),449(6),287(13)	Cyanidin-3-(*p*-coumaroyl-6′-caffeoyl)sophoroside isomer 2 ^A^
**P3**	38.4	522	1005	757(6),535(11),287(11)	Cyanidin-3-(6-*p*-coumaroyl)sophoroside-5-(6-malonyl)glucoside ^A^
**P4**	42.8	522	757	595(100),449(11),287(61)	Cyanidin-3-(6-*p*-coumaroyl)glucoside-5-glucoside ^A^
**P5**	43.2	530	1081	919(15),449(6),287(6)	Cyanidin-3-(6-*p*-coumaroyl-6′-caffeoyl)-5-glucoside isomer1 ^A^
**P6**	43.9	532	1167	919(44),757(5),287(20)	Cyanidin-3-(6-*p*-coumaroyl-6′-caffeoyl)sophoroside-5-(6-malonyl)glucoside isomer 1 ^A^
**P7**	44.6	530	1167	919(27),757(5),287(6)	Cyanidin-3-(6 *p*-coumaroyl-6′-caffeoyl)sophoroside-5-(6-malonyl)glucoside isomer 2 ^A^
**P8**	45.5	530	1081	919(100),449(11),287(20)	Cyanidin-3-(6-*p*-coumaroyl-6′-caffeoyl)sophoroside-5-glucoside isomer2 ^A^
**P9**	45.9	530	1065	903(20),449(5),287(3)	Cyanidin-3-(6,6′-di *p*-coumaroyl)sophoroside-5-glucoside ^A^
**P10**	46.4	526	1151	989(10),903(5),287(5)	Cyanidin-3-(6,6′-di *p*-coumaroyl)sophoroside-5-(6-malonyl)glucoside ^A^
**P11**	47.0	514	1049	887(33),433(9),271(5)	Pelargonidin-3-(6,6′-di *p*-coumaroyl)sophoroside-5-glucoside ^B^
**P12**	48.0	526	1167	1005(63),919(23),449(8),287(13)	Cyanidin-3-(6- *p*-coumaroyl-X-malonyl-6′-caffeoyl)sophoroside-5-glucoside ^A^
**P13**	48.7	530	1151	989(28),449(17),287(5)	Cyanidin-3-(6- *p*-coumaroyl-X-malonyl-6′-*p*-coumaroyl)sophoroside-5-glucoside ^A^

Calibration curves used: ^A^- cyanidin-3-*O*-glucoside (y = 97,787x − 743,469; *R*^2^ = 0.999); ^B^- pelargonidin-3-*O*-glucoside (y = 43,781x − 275,315; *R*^2^ = 0.999).

**Table 2:** The first part describes the experimental design that was applied in this work. The independent variables are presented in coded and natural values. The second part shows the response values for the detected anthocyanin compounds (mg/g E) and extraction yield (%) achieved for all the 28 experimental conditions performed for the HAE by the RSM design.

Five-Level *CCCD* Experimental Design																													
	Runs	1	2	3	4	5	6	7	8	9	10	11	12	13	14	15	16	17	18	19	20	21	22	23	24	25	26	27	28
Coded values	*X*_1_: Time (*t*)	−1	−1	−1	−1	1	1	1	1	1.68	−1.68	0	0	0	0	−1.68	−1.68	−1.68	−1.68	1.68	1.68	1.68	1.68	0	0	0	0	0	0
	*X*_2_: Temp. (*T*)	−1	−1	1	1	−1	−1	1	1	0	0	−1.68	1.68	0	0	−1.68	−1.68	1.68	1.68	−1.68	−1.68	1.68	1.68	0	0	0	0	0	0
	*X*_3_: Solvent (*S*)	−1	1	−1	1	−1	1	−1	1	0	0	0	0	−1.68	1.68	−1.68	1.68	−1.68	1.68	−1.68	1.68	−1.68	1.68	0	0	0	0	0	0
Natural values	*X*_1_: *t* (min)	40.3	40.3	40.3	40.3	99.7	99.7	99.7	99.7	120.0	20.0	70.0	70.0	70.0	70.0	20.0	20.0	20.0	20.0	120.0	120.0	120.0	120.0	70.0	70.0	70.0	70.0	70.0	70.0
	*X*_2_: *T* (°C)	37.2	37.2	72.8	72.8	37.2	37.2	72.8	72.8	55.0	55.0	25.0	85.0	55.0	55.0	25.0	25.0	85.0	85.0	25.0	25.0	85.0	85.0	55.0	55.0	55.0	55.0	55.0	55.0
	*X*_3_: *S* (%)	20.3	79.7	20.3	79.7	20.3	79.7	20.3	79.7	50.0	50.0	50.0	50.0	0.0	100.0	0.0	100.0	0.0	100.0	0.0	100.0	0.0	100.0	50.0	50.0	50.0	50.0	50.0	50.0
**Response Variables for RSM Application**																													
**P1**		3.34	5.61	4.50	5.75	3.41	5.80	3.55	4.96	3.93	5.08	5.05	5.52	3.17	5.78	1.96	6.36	4.56	5.83	2.27	5.86	1.42	5.11	4.93	4.91	5.34	5.35	5.24	4.84
**P2**		2.47	4.24	2.64	4.15	2.38	4.27	2.26	3.72	2.55	3.32	3.15	2.92	2.31	5.31	1.78	4.85	2.50	4.11	1.83	4.22	1.45	3.83	3.43	3.54	3.47	3.48	3.44	3.29
**P3**		3.94	5.53	4.71	5.37	3.60	6.52	3.90	5.78	3.93	4.89	5.29	5.74	2.81	5.13	2.10	4.25	2.98	3.16	1.59	4.63	1.48	5.42	6.33	7.04	6.70	6.71	6.94	6.67
**P4**		2.95	5.68	2.82	5.05	2.93	6.60	2.71	5.01	3.70	4.26	4.98	4.97	1.66	4.60	1.79	5.27	1.47	4.67	1.59	4.61	1.39	5.12	4.59	4.57	4.78	4.79	4.40	4.42
**P5**		7.61	13.13	8.39	13.66	7.30	13.00	8.15	13.66	10.54	12.68	15.27	17.09	2.64	9.81	1.87	8.32	1.47	10.19	1.59	8.67	1.39	11.07	16.99	16.40	17.62	17.66	15.93	16.40
**P6**		3.47	5.69	3.78	5.34	3.48	4.99	3.52	5.02	3.91	5.09	5.68	5.74	1.70	4.01	1.74	4.70	1.47	3.76	1.59	3.99	1.37	3.25	6.35	6.10	6.62	6.64	6.34	6.31
**P7**		2.52	4.25	2.62	3.98	2.43	3.59	2.41	3.62	3.12	3.52	3.80	3.64	1.75	4.07	1.74	4.83	1.91	3.98	1.59	4.07	1.37	3.28	3.73	3.75	3.85	3.86	3.69	3.69
**P8**		3.53	5.93	3.67	5.39	3.40	6.22	3.36	5.03	3.95	5.09	5.42	5.28	1.96	3.84	1.85	4.65	1.47	3.82	1.59	3.96	1.37	3.49	5.86	5.64	5.88	5.89	6.01	5.57
**P9**		5.10	8.37	5.33	7.82	4.78	8.55	4.94	7.82	5.46	7.15	8.25	8.59	2.07	5.44	1.94	5.27	1.47	4.98	1.59	4.70	1.40	5.41	9.99	9.52	10.25	10.27	9.49	9.71
**P10**		14.92	21.10	16.36	24.14	14.84	18.68	16.01	24.14	21.50	23.40	26.73	30.48	6.96	14.17	2.29	9.46	2.35	13.48	1.59	10.19	1.43	17.38	32.57	32.82	33.99	34.06	32.48	33.27
**P11**		10.18	14.08	13.19	15.07	9.34	12.26	10.90	13.27	12.83	17.10	16.97	19.23	5.98	5.71	2.31	5.13	5.67	5.26	1.27	4.82	1.16	5.32	20.04	19.79	20.91	20.95	21.22	19.94
**P12**		2.91	4.60	3.27	4.31	2.66	4.10	2.76	3.81	3.22	4.13	4.19	4.26	2.20	4.09	1.92	4.80	2.74	4.17	1.59	4.29	1.41	3.54	3.93	3.80	3.83	3.84	3.94	3.83
**P13**		4.84	7.26	5.77	6.99	4.30	7.53	4.89	6.72	5.63	7.31	7.30	7.85	2.73	4.57	2.08	4.75	3.32	4.12	1.59	4.08	1.40	4.15	8.06	8.08	8.34	8.36	8.75	8.38
TAC		67.78	105.46	77.06	107.01	64.86	102.12	69.37	102.56	84.25	103.00	112.07	121.30	37.96	76.52	25.38	72.65	33.37	71.53	21.22	68.10	18.04	76.37	116.79	115.97	111.59	111.85	117.87	116.32
Yield		36.35	28.58	38.26	31.41	36.08	29.95	39.41	32.14	38.12	37.53	34.75	37.90	38.84	18.08	35.62	13.22	38.27	17.80	35.42	16.52	41.00	20.24	35.68	34.54	35.68	35.61	35.54	35.40

**P**: anthocyanin compound; TAC: Total anthocyanin content.

**Table 3:** Estimated coefficients and *R*^2^ determined for the models obtained for individual and grouped anthocyanin compounds and extraction yield (Table 3), and optimal HAE conditions and response values.

Response variables	Fitting Coefficients Obtained after Applying the Second-Order Polynomial Equation with Interactive Terms														Optimal Processing Conditions and Response Values
	Intercept	Linear Effect			Quadratic Effect			Interactive Effect			*R* ^2^	*t* (min)	*T* (°C)	*S* (%)	*Optimum*
	*b*_0_	*b*_1_ (*t*)	*b*_2_ (*T*)	*b*_3_ (*S*)	*b*_11_ (*t*^2^)	*b*_22_ (*T*^2^)	*b*_33_ (*S*^2^)	*b*_12_ (*tT*)	*b*_13_ (*tS*)	*b*_23_ (*TS*)					
**P1**	5.06 ± 0.15	−0.28 ± 0.09	ns	0.92 ± 0.09	−0.16 ± 0.11	ns	−0.17 ± 0.11	−0.17 ± 0.06	0.07 ± 0.06	−0.15 ± 0.06	0.9441	81.06 ± 2.08	20.00 ± 1.73	100.00 ± 1.58	6.56 ± 0.31
**P2**	3.37 ± 0.10	−0.15 ± 0.06	ns	0.76 ± 0.06	−0.20 ± 0.07	ns	0.11 ± 0.07	−0.04 ± 0.00	ns	−0.07 ± 0.04	0.9556	64.04 ± 5.07	20.00 ± 0.43	100.00 ± 9.11	5.15 ± 0.36
**P3**	31.23 ± 1.68	ns	0.96 ± 0.95	ns	−2.14 ± 1.15	ns	−5.40 ± 1.15	ns	0.98 ± 0.68	ns	0.9359	70.00 ± 3.94	90.00 ± 6.07	50.00 ± 3.80	32.85 ± 2.47
**P4**	4.65 ± 0.19	ns	−0.20 ± 0.11	1.06 ± 0.11	−0.11 ± 0.01	ns	−0.41 ± 0.13	ns	ns	ns	0.9225	70.00 ± 5.49	20.00 ± 1.94	88.44 ± 5.39	5.67 ± 1.05
**P5**	15.59 ± 0.62	ns	0.33 ± 0.22	2.42 ± 0.35	−0.95 ± 0.42	ns	−2.85 ± 0.42	ns	ns	ns	0.9449	70.00 ± 1.26	90.00 ± 5.25	62.60 ± 5.86	16.66 ± 1.76
**P6**	5.90 ± 0.27	−0.16 ± 0.15	−0.10 ± 0.01	0.74 ± 0.15	−0.31 ± 0.18	ns	−0.89 ± 0.18	ns	ns	ns	0.9336	62.47 ± 1.24	20.00 ± 0.31	62.27 ± 3.93	6.24 ± 0.56
**P7**	3.63 ± 0.08	−0.16 ± 0.05	−0.09 ± 0.05	0.70 ± 0.05	−0.08 ± 0.06	ns	−0.23 ± 0.06	ns	ns	−0.07 ± 0.03	0.9701	40.93 ± 1.60	20.00 ± 1.49	100.00 ± 6.12	4.59 ± 0.34
**P8**	5.59 ± 0.18	−0.13 ± 0.10	−0.14 ± 0.10	0.77 ± 0.10	−0.23 ± 0.12	ns	−0.80 ± 0.12	ns	ns	ns	0.9456	61.55 ± 4.64	20.00 ± 1.03	64.30 ± 1.89	6.03 ± 0.55
**P9**	9.06 ± 0.43	ns	−0.12 ± 0.02	1.15 ± 0.24	−0.62 ± 0.30	ns	−1.52 ± 0.30	ns	ns	ns	0.9286	70.00 ± 1.16	20.00 ± 1.24	61.18 ± 2.85	9.47 ± 1.32
**P10**	29.87 ± 1.32	ns	0.85 ± 0.74	2.89 ± 0.74	−2.07 ± 0.91	ns	−6.27 ± 0.91	ns	ns	ns	0.9377	70.00 ± 1.42	90.00 ± 2.46	56.85 ± 0.94	31.63 ± 2.42
**P11**	8.93 ± 0.25	−0.32 ± 0.14	−0.12 ± 0.14	0.63 ± 0.14	−0.47 ± 0.17	ns	−1.64 ± 0.17	ns	ns	−0.10 ± 0.01	0.9359	59.99 ± 5.19	20.00 ± 0.59	57.18 ± 0.16	9.28 ± 0.38
**P12**	3.91 ± 0.06	−0.22 ± 0.03	−0.03 ± 0.03	0.65 ± 0.03	−0.06 ± 0.04	ns	−0.25 ± 0.04	−0.05 ± 0.02	ns	−0.09 ± 0.02	0.9757	36.49 ± 0.30	20.00 ± 1.21	98.54 ± 9.29	4.71 ± 0.20
**P13**	7.93 ± 0.20	−0.26 ± 0.11	−0.16 ± 0.11	0.73 ± 0.11	−0.37 ± 0.03	ns	−1.37 ± 0.13	ns	0.09 ± 0.08	−0.10 ± 0.01	0.9451	60.74 ± 0.00	20.00 ± 1.30	59.46 ± 0.04	8.38 ± 0.29
TAC	109.78 ± 2.73	−1.93 ± 1.54	1.07 ± 0.32	14.30 ± 1.54	−6.20 ± 1.87	ns	−17.00 ± 1.87	ns	ns	ns	0.9577	65.37 ± 3.62	90.00 ± 1.17	62.50 ± 4.24	114.74 ± 0.58
Yield	36.43 ± 1.46	0.49 ± 0.88	1.19 ± 0.87	−5.56 ± 0.87	ns	ns	−3.09 ± 0.84	ns	ns	ns	0.9592	120.00 ± 2.62	90.00 ± 7.72	23.23 ± 0.91	41.77 ± 1.59

ns: non-significant coefficient; *R*^2^: Correlation coefficient; **P**: anthocyanin compound; TAC: total anthocyanin content.

**Table 4:** Mathematical models produced after fitting Equation (1) to the data set (individual and grouped values).

Anthocyanin Compounds	Equations	Equation Numbers
**P1**	$Y_{P1}^{}=5.06-0.28t+\;0.92S-0.16t^{2}-0.17S^{2}-0.17tT+0.07tS-0.15TS$	Equation (2)
**P2**	$Y_{P2}^{}=3.37-0.15t+\;0.76S-0.20t^{2}-0.11S^{2}-0.04tT-0.07TS$	Equation (3)
**P3**	$Y_{P3}^{}=31.23+\;0.96T-2.14t^{2}-5.40S^{2}+0.98tS$	Equation (4)
**P4**	$Y_{P4}^{}=4.65-0.20T+\;1.06S-0.11t^{2}-0.41S^{2}$	Equation (5)
**P5**	$Y_{P5}^{}=15.59+0.33T+\;2.42S-0.95t^{2}-2.85S^{2}$	Equation (6)
**P6**	$Y_{P6}^{}=5.90-0.16t-0.10T+\;0.74S-0.31t^{2}-0.89S^{2}$	Equation (7)
**P7**	$Y_{P7}^{}=3.63-0.16t-0.09T+\;0.70S-0.08t^{2}-0.23S^{2}-0.07TS$	Equation (8)
**P8**	$Y_{P8}^{}=5.59-0.13t-0.14+\;0.77S-0.23t^{2}-0.80S^{2}$	Equation (9)
**P9**	$Y_{P9}^{}=9.06-0.12T+\;1.15S-0.62t^{2}-1.52S^{2}$	Equation (10)
**P10**	$Y_{P10}^{}=29.87+0.85T+\;2.89S-2.07t^{2}-6.27S^{2}$	Equation (11)
**P11**	$Y_{P11}^{}=8.93-0.32t-0.12+\;0.63S-0.47t^{2}-1.64S^{2}-0.10TS$	Equation (12)
**P12**	$Y_{P12}^{}=3.91-0.22t-\;0.03T+0.65S-0.06t^{2}-0.25S^{2}-0.05tT-0.09TS$	Equation (13)
**P13**	$Y_{P13}^{}=7.93-0.26t-0.16T+\;0.73S-0.37t^{2}-1.37S^{2}+0.09tS-0.10TS$	Equation (14)
TAC	$Y_{TAC}^{}=109.78-1.93t+\;1.07T+14.30S-6.20t^{2}-17.00S^{2}$	Equation (15)
Yield	$Y_{Yield}^{}=36.43+0.49t+\;1.19T-5.56S-3.09S^{2}$	Equation (16)

**Table 5:** Maximum response values of each anthocyanin compound and their values at the optimal processing conditions of the other compounds presented in Table 3.

**(A) Maximum Response Values (mg/g of Extract) of the Individual Anthocyanin Compounds**													
Peak:	**P1**	**P2**	**P3**	**P4**	**P5**	**P6**	**P7**	**P8**	**P9**	**P10**	**P11**	**P12**	**P13**
Optimum:	6.56	5.15	32.85	5.67	16.66	6.24	4.59	6.03	9.47	31.63	9.28	4.71	8.38
**(B) Values of each Anthocyanin Compound at the Optimal Conditions of the other Compounds**													
	**P1**	**P2**	**P3**	**P4**	**P5**	**P6**	**P7**	**P8**	**P9**	**P10**	**P11**	**P12**	**P13**
**P1**	1	0.99	0.77	0.96	0.81	0.84	0.95	0.85	0.83	0.79	0.81	0.94	0.82
**P2**	0.99	1	0.65	0.91	0.71	0.73	0.98	0.74	0.72	0.68	0.70	0.95	0.71
**P3**	0.45	0.42	1	0.63	0.97	0.87	0.33	0.85	0.88	0.99	0.88	0.33	0.88
**P4**	0.99	0.99	0.76	1	0.83	0.94	0.97	0.95	0.94	0.80	0.92	0.97	0.93
**P5**	0.65	0.66	0.97	0.80	1	0.93	0.61	0.93	0.93	0.99	0.92	0.61	0.93
**P6**	0.75	0.77	0.92	0.89	0.94	1	0.74	1.00	1.00	0.94	1.00	0.75	1.00
**P7**	0.97	0.99	0.76	0.97	0.80	0.90	1	0.91	0.88	0.79	0.87	1.00	0.88
**P8**	0.79	0.81	0.89	0.91	0.92	1.00	0.79	1	1.00	0.91	0.99	0.80	1.00
**P9**	0.72	0.72	0.93	0.86	0.96	1.00	0.66	0.99	1	0.95	0.99	0.66	0.99
**P10**	0.48	0.49	0.99	0.69	0.99	0.90	0.43	0.89	0.91	1	0.90	0.44	0.90
**P11**	0.61	0.63	0.94	0.80	0.93	0.99	0.61	0.99	0.99	0.94	1	0.63	1.00
**P12**	0.97	0.99	0.82	0.98	0.85	0.91	1.00	0.92	0.90	0.84	0.89	1	0.90
**P13**	0.69	0.70	0.91	0.84	0.91	1.00	0.67	1.00	1.00	0.92	1.00	0.68	1

**Table 6:** Amount of anthocyanins (cyanidin and pelargonidin derivatives) and color parameters under optimal conditions (mean ± SD).

Quantification (mg/g E)	L*	a*	b*	Conversion Color to RGB Values
115.4 ± 0.4	20.5 ± 0.5	33.0 ± 0.1	8.2 ± 0.4	

L* lightness; a* chromatic axis from green (−) to red (+); b* chromatic axis from blue (−) to yellow (+).

**Table 7:** Antibacterial activity (MIC and MBC, mg/mL) and antifungal activity (MIC and MFC, mg/mL) of the anthocyanins rich extract obtained under optimal extraction conditions.

**Antibacterial Activity**							
		***B.c.***	***S.a.***	***L.m.***	***E.c.***	***En.cl.***	***S.t.***
Anthocyanins rich extract	MIC	0.037	0.075	0.05	0.037	0.075	0.15
	MBC	0.075	0.15	0.075	0.075	0.15	0.30
Streptomycin ^(1)^	MIC	0.10	0.04	0.20	0.20	0.20	0.20
	MBC	0.20	0.10	0.30	0.30	0.30	0.30
Ampicillin ^(1)^	MIC	0.25	0.25	0.40	0.40	0.25	0.75
	MBC	0.40	0.45	0.50	0.50	0.50	1.20
**Antifungal Activity**							
		***A.fun.***	***A.o.***	***A.n.***	***P.f.***	***P.o***	***P.v.c.***
Anthocyanins rich extract	MIC	0.037	0.002	0.075	0.075	0.30	0.30
	MFC	0.075	0.075	0.15	0.15	0.45	0.45
Ketoconazole ^(1)^	MIC	0.25	0.20	0.20	0.20	2.50	0.20
	MFC	0.50	0.50	0.50	0.50	3.50	0.30
Bifonazole ^(1)^	MIC	0.15	0.10	0.15	0.20	0.20	0.10
	MFC	0.20	0.20	0.20	0.25	0.25	0.20

^(1)^ Positive controls. *B.c.: Bacillus cereus; S.a.: Staphylococcus aureus; L.m.: Listeria monocytogenes; E.c.: Escherichia coli; En.cl.: Enterobacter cloacae; S.t.: Salmonella typhimurium; A.fum.: Aspergillus fumigatus; A.o.: Aspergillus ochraceus; A.n.: Aspergillus niger; P.f.: Penicillium funiculosum; P.o.: Penicillium ochrochloron; P.v.c.: Penicillium verrucosum var. cyclopium*. MIC—minimum inhibitory concentration; MBC—minimum bactericidal concentration; MFC—minimum fungicidal concentration.

**Table 8:** Cytotoxic activity of the anthocyanins rich extract obtained under optimal extraction conditions (mean ± SD).

Tumor Cell Lines	Concentrations (GI_50_ Values, µg/mL)
MCF-7 (breast carcinoma)	>400
NCI-H460 (lung carcinoma)	>400
HeLa (cervical carcinoma)	213 ± 9
HepG2 (hepatocellular carcinoma)	198 ± 9
**Non-Tumour Cells**	
PLP2 (non-tumor porcine liver primary cells)	>400

GI_50_ values - concentration that inhibited 50% of cell growth. Ellipticin GI_50_ (positive control): 1.21 μg/mL (MCF-7), 1.03 μg/mL (NCI-H460), 0.91 μg/mL (HeLa), 1.10 μg/mL HepG2) and 2.29 μg/mL (PLP2).
